# Reward and punisher experience alter rodent decision-making in a judgement bias task

**DOI:** 10.1038/s41598-020-68737-1

**Published:** 2020-07-16

**Authors:** Vikki Neville, Jessica King, Iain D. Gilchrist, Peter Dayan, Elizabeth S. Paul, Michael Mendl

**Affiliations:** 1 Bristol Veterinary School, University of Bristol, Bristol, BS40 5DU UK; 2 Animal Sciences Department, University of Florida, Florida, FL 32608 USA; 3 School of Psychological Science, University of Bristol, Bristol, BS8 1TU UK; 4 Max Planck Institute for Biological Cybernetics, Max Plank-Ring 8, 72076 Tübingen, Germany

**Keywords:** Animal behaviour, Emotion, Motivation, Reward, Computational neuroscience

## Abstract

The influence of affective states on decision-making is likely to be complex. Negative states resulting from experience of punishing events have been hypothesised to generate enhanced expectations of future punishment and ‘pessimistic’/risk-averse decisions. However, they may also influence how decision-outcomes are valued. Such influences may further depend on whether decisions at hand are germane to the rewards or punishers that induced the affective state in the first place. Here we attempt to dissect these influences by presenting either many or few rewards or punishers of different types (sucrose vs air-puff; 50 kHz vs 22 kHz ultrasonic vocalisations) to rats, and investigating their subsequent decisions in a judgement bias task that employed sucrose and air-puff as decision outcomes. Rats that received many sucrose pellets prior to testing were more risk-averse than those receiving many air-puffs. Ultrasonic vocalisations did not alter decision-making. Computational analysis revealed a higher weighting of punishers relative to rewards (in agreement with findings from a separate behavioural task) and a bias towards the risk-averse response following pre-test sucrose compared to pre-test air-puff. Thus, in this study reward and punisher manipulation of affective state appeared to alter decision-making by influencing both expectation and valuation of decision-outcomes in a domain-specific way.

## Introduction

Certain features of negatively and positively valenced affective states are phylogenetically widespread^1–5^, suggesting that affect confers an evolutionary advantage. A prominent hypothesis is that affect, having both transient and longer-lasting components, provides information about the ongoing state of the environment and an animal’s situation within it, thereby allowing adaptive behavioural decision-making^2,4,6,7^. Sensory information can be noisy and there may be some degree of overlap between cues that signal the arrival of rewards or punishers and background noise. When decisions need to be made about how best to respond to such complex and sometimes ambiguous information to maximise fitness, affective states may be valuable^8^. For example, a rustling noise could either signal an approaching predator or just be the sound of wind moving vegetation. In such a situation a prey animal must decide whether to flee, which would be energetically costly but avoid the risk of predation, or to ignore the noise and conserve energy but risk attack. Repeated prior encounters with aversive and punishing stimuli (things that animals work to avoid) are suggested to lead to a negative affective state (see operational definitions in Rolls 2005^9^; Paul and Mendl 2018^10^; Mendl and Paul 2020^8^) which decrease an individual’s threshold for responding to future potentially threatening signals and hence favours safety-first, risk-averse decisions under ambiguity^3,4^. However, an animal that has experienced few punishing experiences, and is consequently in a less negative state, might have a higher response threshold for potential threats^2–4^. Similarly, such affective states might oppositely modulate an individual’s threshold for responding to potential rewards.

In essence, functional explanations of affect such as these suggest that ‘emotions’ and ‘moods’ encode information about past reward and punisher experience and hence act as Bayesian-like priors on the probability of rewarding and punishing decision-outcomes, thus allowing optimisation of future reward acquisition and punisher avoidance^2,4,6,7^. However, it is also likely that past experience and resulting affective states (e.g. depression-related anhedonia^11–13^) influence not just estimates of the *probability* of decision outcomes but also their *value*. The influence of past experience on reward and punisher valuation might have opposite effects on decision-making compared with its influence on reward and punisher probability estimation. For example, inhabiting an environment in which threats are frequent might increase an individual’s estimation of the probability of punishers (resulting in ‘pessimistic’/risk-averse decision-making) but also increase their valuation of food (resulting in more ‘optimistic’/risk-seeking decision-making) as maintaining the high energy reserves needed to evade predation becomes more important^14^. Likewise, an environment that is abundant in food might lead to both an increased probability estimation of rewards (‘optimistic’/risk-seeking decision-making) but a decreased valuation of food (‘pessimistic’/risk-averse decision-making) via satiation.

Furthermore, a key component of functional hypotheses of affect is the notion of ‘domain generality’; the proposal that the affective system encapsulates an individual’s general experiences of reward and punishment (and the prevalence and severity of these), regardless of the specific types of rewards and punishers concerned. Thus it is hypothesised that affective states or moods reflect a kind of meta-learning, not just about the probability and value of each individual punisher and reward they are likely to come across in their environment, but about rewards and punishers in general. A broadly negative state would be expected to arise if punishers are common and severe while rewards are sparce and slight, and to drive more cautious and risk-averse decisions across domains. This would be adaptive if such domain-general states and effects reflect actual correlations between different types of reward and different types of punishers in an animal’s everyday life.

The relationship between affect and decision-making may thus be complex. The way in which decisions are influenced is likely to depend on whether and how affective states alter perceived *likelihoods* and *values* of different decision outcomes, and also on whether they have a *domain-general* influence across all reward and punisher types, or exert a *domain-specific* effect on decisions that are directly related to the rewards and punishers which induced the current affective state. Disentangling these possibilities has implications not just for our understanding of the functional role of affective states, but also for the utility of measuring changes in decision-making as markers of affective valence^8,15–17^.

The aim of this paper is to address these issues using a paradigm developed specifically to evaluate the influence of affective states on decision-making under ambiguity. The judgement bias task is a widely used translational paradigm for the investigation of affective state, and hence welfare, across numerous species including rats, starlings, sheep and humans^15–23^. In a common variant of this task, an individual is trained to choose between two different actions in response to two sensory stimuli that occupy different ends of a sensory continuum (e.g. high and low pitched tones). One action is ‘safe’, leading to neither reward nor punishment for either stimulus. The other action is ‘risky’; whether the animal receives a reward or punishment following its execution depends on the previous discriminatory sensory stimulus. During training, animals learn to accurately discriminate between cues that predict rewards and those that predict punishment by performing one of the two alternative responses. During testing, they are also required to take one of the actions in response to occasional new, intermediate, ambiguous sensory stimuli. Individuals who are more risk-seeking or ‘optimistic’ (less likely to execute the ‘safe’ action) when presented with intermediate ambiguous stimuli are thought to be experiencing a more positively-valenced affective state compared with those that are more risk-averse or ‘pessimistic’ (i.e. more likely to execute the ‘safe’ action) . A recent meta-analysis indicates general support for this hypothesis, but significant heterogeneity in findings^17^. One possibility is that the influence of affective manipulations alters both the perceived likelihood and valuation of decision-outcomes. Dissecting any such effects should thus aid in the interpretation of findings, and help explain contradictory results.

In this study we used the judgement bias task in rats to investigate three questions. (1) Are individuals more risk-seeking in the judgement bias task following experience of rewards compared to punishers, and is this dependent on reward and punisher prevalence? (2) Does this effect depend on the extent to which the rewards and punishers are ‘task-specific’ (i.e. the same as those used in the judgement bias task), or can experience of rewards and punishers in one domain influence decision-making in another domain? (3) Does risk-seeking or risk-averse behaviour in these situations arise from modulation of the perceived likelihood and/or value of rewarding and punishing outcomes?

To answer these questions, we delivered either many or few rewards or punishers to rats immediately prior to a judgement bias task. The rewards or punishers were either task-specific (sucrose pellet vs air-puff) or task irrelevant (50 kHz ‘positive’ vs 22 kHz ‘negative’ ultrasonic vocalisations^24^). To investigate whether these stimuli influenced decision-making by altering perceived likelihood or valuation of decision outcomes, judgement bias data were analysed using computational modelling that allowed the influence of these parameters to be modelled. Independently, we also studied the influence of the same stimuli on a progressive ratio lever-pressing task to investigate how they altered motivation to work for (valuation of) the sucrose reward decision-outcome used in the judgement bias task.

## Results

We administered an automated judgement bias task^25^ in which rats responded on each trial to stimuli (auditory tones ranging from 2 to 8 kHz) that either unambiguously (2 kHz or 8 kHz) or ambiguously (intermediate tones) predicted rewards (sucrose pellet) and punishers (air puff). Before completing each test session, animals were exposed to one of eight different environments in a randomised, factorial, repeated measures design. In each pre-test environment, three factors were systematically varied: *valence* (reward versus punisher), *task-specificity* (specific: rewards and punishers the same as those used in the subsequent judgement bias task (sucrose or air puff) versus non-specific: rewards and punishers not the same as those used in the judgement bias task (auditory playback of ‘positive’ and ‘negative’ ultrasonic vocalisations)) and *prevalence* of rewards and punishers (low versus high). We employed a model-agnostic statistical analysis to summarise the raw data and we fitted a Bayesian decision-theoretic model^21^ which allowed us to estimate parameters relating to the decision-making process, namely reward valuation and probability estimation. A progressive ratio lever pressing task for sucrose pellet rewards was also administered to the rats. This provided an additional behavioural measure of reward valuation to complement the measure of reward and punisher valuation obtained through the model-dependent analysis of the judgement bias data.

### Statistical analysis: judgement bias data

**Figure 1:**
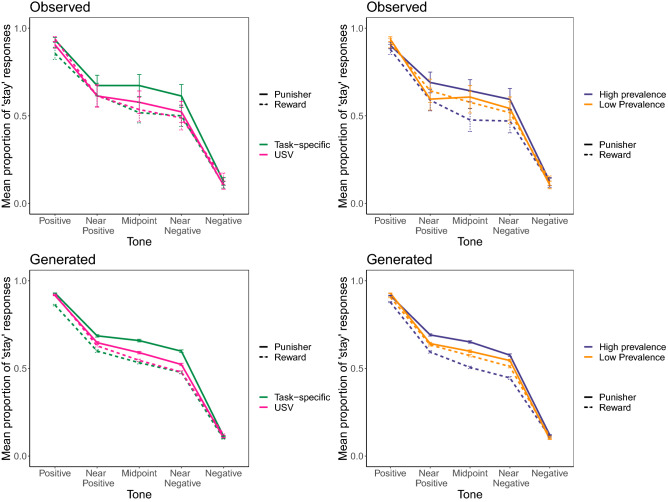
Mean proportion of ‘risk-seeking’ (stay) responses in both the observed and model-generated data split by tone presented following experience of high or low prevalence rewards or punishers and following experience of rewards or punishers that were either task specific or USVs. The top plots show the observed data while the bottom plots show the data generated using the model. The left-hand plots show the data split by task-specificity and manipulation valence (i.e. sucrose—green dashed line; air-puffs—green solid line; 50 kHz USVs—pink dashed line; 22 kHz USVs—pink solid line) and the right-hand plots show the data split by manipulation prevalence and valence (i.e. high prevalence rewards—purple dashed line; high prevalence punishers—purple solid line; low prevalence rewards—orange dashed line; and low prevalence punishers—orange solid line). Error bars represent one standard error.

Stimulus tone was a significant predictor of response, indicating that rats were able to discriminate between the stimuli used in the judgement bias task (Likelihood ratio test, LRT = 3,628.970, $\hbox{p}<0.001$). Rats tended to be more risk-seeking when the outcome of the previous trial was more aversive (LRT = 3.023, p = 0.082) and were significantly more risk-seeking when the 2 kHz compared with the 8 kHz tone was rewarded (LRT = 14.331, $\hbox{p}<0.001$). Manipulation valence (LRT = 2.300, p = 0.129), task-specificity (LRT = 0.109, p = 0.741), prevalence (LRT = 0.762, p = 0.383), and number of trials completed (LRT = 2.409, 0.121) were not significant as main effects. However, there was a marginally non-significant interaction between valence and prevalence (Fig. 1: LRT = − 2.294, p = 0.075) and a significant interaction between valence and task-specificity (Fig. 1: LRT = 2.753, p = 0.021). Post-hoc analyses indicated that the difference in judgement bias was greatest between rats exposed to the high prevalence rewards compared to high prevalence punishers, and only for task-specific rewards compared to task-specific punishers (Table 1). The interaction between valence and stimulus was non-significant (LRT = 5.523, p = 0.238) as was the interaction between valence, prevalence, and task-specificity (LRT = 0.794, p = 0.672). In sum, rats were most risk-seeking following delivery of pre-test air-puffs at a high prevalence and most risk-averse following delivery of pre-test sucrose at a high prevalence.

**Table 1:** Results of the post-hoc analyses of the interaction between manipulation prevalence and valence, and manipulation specificity and valence on judgement bias data.

Contrast	z-value	p-value
Task-specific vs. non-specific rewards	− 1.429	0.417
Task-specific vs. non-specific punishers	2.093	0.120
Non-specific rewards vs. punishers	0.751	0.845
**Task-specific rewards vs. punishers**	**2.753**	**0.021**
High vs. low prevalence rewards	2.018	0.142
High vs. low prevalence punishers	− 0.598	0.914
Low prevalence rewards vs. punishers	− 0.328	0.984
High prevalence rewards vs. punishers	2.294	0.075

Bold values highlight statistically significant results.

To investigate whether there were treatment differences in rats’ behaviour following sucrose or air-puff delivery which might indicate satiation to sucrose or habituation to the air-puffs, we analysed the rats’ latency to initiate the next trial following these outcomes as a proxy for sucrose consumption speed and air-puff recovery time. The duration of self-determined inter-trial intervals following sucrose delivery was not affected by the interaction between manipulation valence, prevalence, and task-specificity (LRT = 0.210, p = 0.647), between manipulation valence and task-specificity (LRT = 1.856, p = 0.173) or between manipulation valence and prevalence (LRT = 0.088, p = 0.767). Similarly, the interactions between manipulation valence, prevalence, and task-specificity (LRT = 0.889, p = 0.346), between manipulation valence and task-specificity (LRT = 1.276, p = 0.259), and between manipulation valence and prevalence (LRT = 0.515, p = 0.473) were not significant predictors of the duration of the self-determined inter-trial interval following air-puff delivery.

### Statistical analysis: progressive ratio lever pressing data

**Figure 2:**
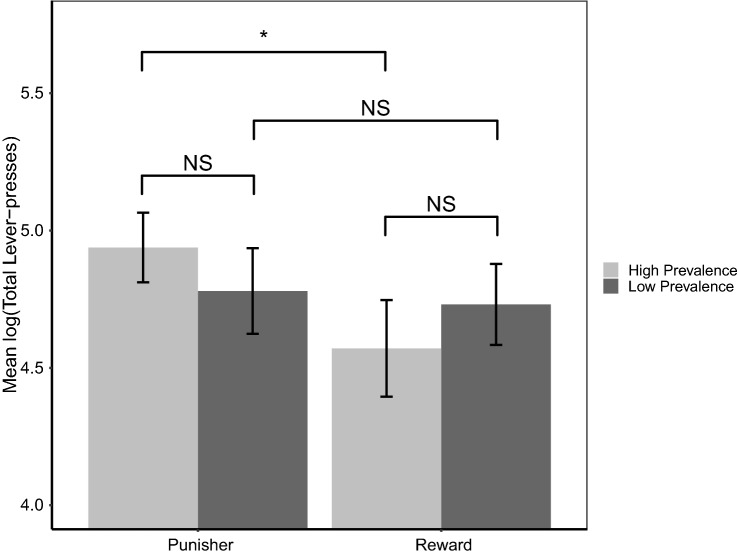
The effect of manipulation valence and prevalence on the log-transformed number of lever presses collapsed across task-specificity. Error bars represent one standard error.

In the progressive ratio lever pressing task, rats pressed the lever more when they had experienced pre-test punishers than pre-test rewards (mean number of lever presses ± standard error, SE: reward: $159\pm 20.4$, punishers: $167\pm 14.1$, LRT = 4.944, p = 0.026). The numerical prevalence of pre-test rewards and punishers ($\hbox{LRT}<0.001$, $\hbox{p}=0.993$), or task-specificity (LRT = 1.927, p = 0.165) did not significantly predict the number of lever presses. There was a marginally non-significant interaction between manipulation valence and prevalence (Fig. 2: LRT = 3.003, p = 0.083). While there was a significant difference between rats that experienced high prevalence rewards and high prevalence punishers (mean number of lever presses ± SE: reward: $159\pm 32.1$, punishers: $174\pm 20.2$, LRT = − 2.913, p = 0.013), no significant difference was observed between rats that experienced rewards and punishers at a low prevalence (LRT = 0.421, p = 0.967), or between rats experiencing rewards at a high or low prevalence (LRT = 1.248, p = 0.533), or punishers at a high or low prevalence (LRT = − 1.247, p = 0.534). There was no significant interaction between manipulation valence and task-specificity (sucrose/air puffs versus USVs) (LRT =  0.074, p = 0.785), or between manipulation valence, task-specificity, and prevalence (LRT = 0.312, p = 0.856). Thus, rats were most motivated to obtain sucrose when they had received pre-test punishers at a high prevalence and least motivated to obtain sucrose when they had received pre-test rewards at a high prevalence, irrespective of reward and punisher type.

### Model-dependent analysis

**Figure 3:**
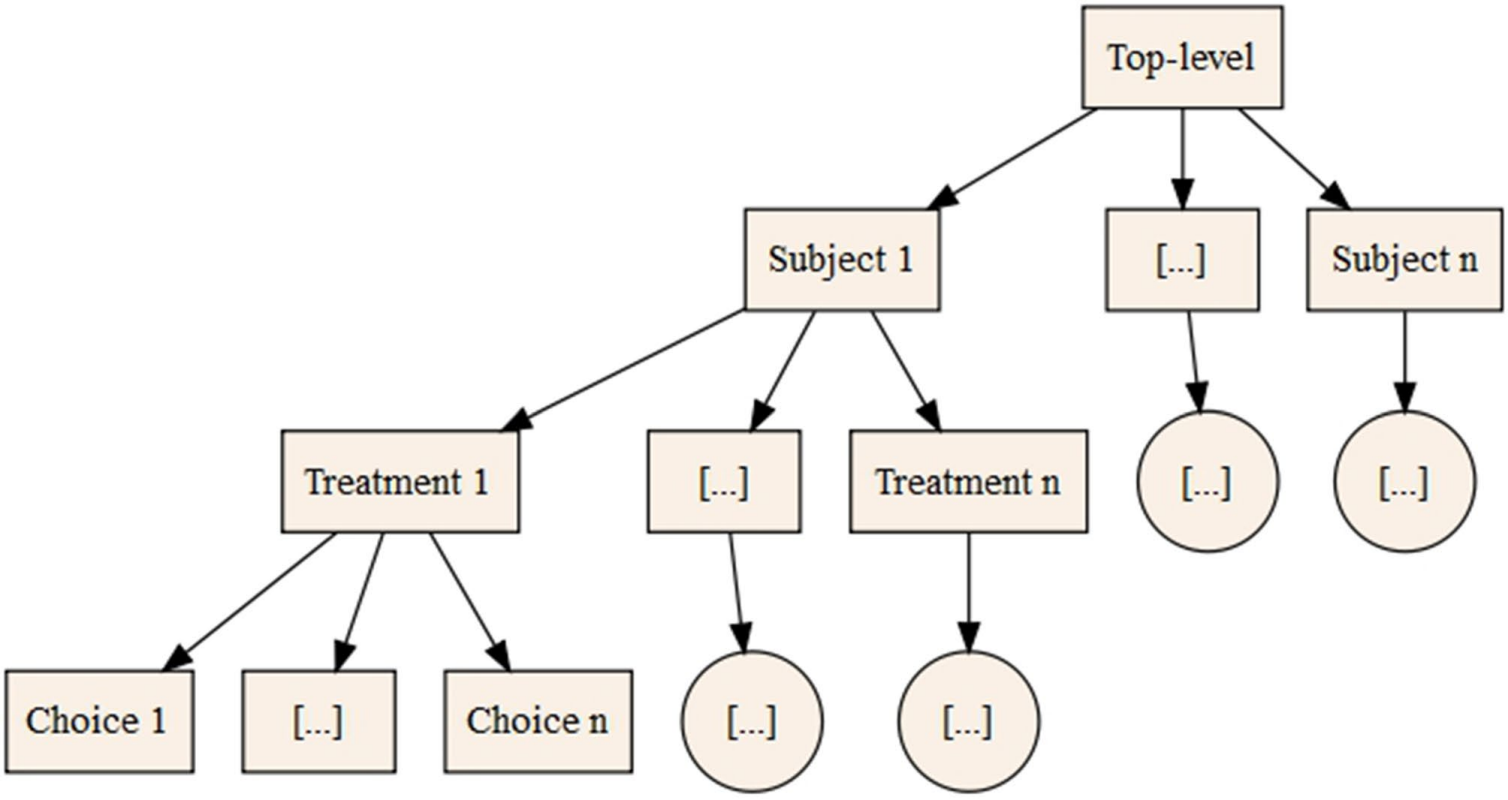
The hierarchical structure of the model-dependent analysis: each choice is determined by the treatment-level parameter distributions, which are determined by the subject-level parameter distributions, and these subject-level parameter distributions are determined by a top-level parameter distribution.

We fitted a Bayesian-decision theoretic model to the judgement bias decision data, testing which of the complete collection of parameters were required to explain the data most parsimoniously. The full model included six parameters: bias ($\delta$), differential sensitivity to outcome punishment versus reward ($C_{p/r}$), a slope parameter which accounted for differences in the rats’ ability to discriminate between the stimuli ($\sigma$), a lapse rate which accounted for errors independent of the presented stimulus ($\lambda$), and parameters which allowed decision-making to vary according to the previous outcome during the test session ($\omega$) and according to which of the two reference stimuli were rewarded and punished ($\beta$). The latter two parameters allowed us to account for effects observed in the model-agnostic analysis. Each model was fitted using a four-level hierarchical Bayesian random effects analysis with a single top level empirical prior distribution of the model parameters across all subjects and within each subject (see Fig. 3). Therefore, the model fitting procedure was blind to the existence of the different conditions. Unlike the remaining parameters, $\beta$ was fitted at three levels instead of four levels, this was because we anticipated that intrinsic preference for one or other tone would be constant within a subject (noting that which tone was rewarded was also constant for a subject).

When penalizing model complexity (using the integrated Bayesian information criterion; iBIC), the most parsimonious model included all parameters except $\delta$ ($C_{p/r}$, $\lambda$, and $\sigma$, $\omega$, and $\beta$; Table 2). Yet, the estimates from the full model revealed that, across all conditions and subjects, both the log-transformed parameter estimates of $C_{p/r}$ (mean ± SE: $-1.103\pm 0.023$, $\hbox{p}<0.001$) and the estimates of $\delta$ (mean $\pm$ SE: −0.100 $\pm$ 0.045, p = 0.026) were significantly less than zero. Hence, we opted to use the full model for further analysis to gain a more complete picture of the potential decision processes contributing to treatment differences in judgement bias. This suggests that general risk-seeking behaviour observed in the rats during the judgement bias task is attributed to greater sensitivity to within-test rewards relative to within-test punishers, and that this effect may be attenuated by an overall bias towards the risk-averse response. The estimates of $\omega$ (mean ± SE: $-0.189\pm 0.045$, $\hbox{p}<0.001$) and $\beta$ (mean ± SE: $0.569\pm 0.032$, $\hbox{p}<0.001$) were also significantly less than zero, suggesting that, as was also observed in the model-agnostic analysis, rats were more risk-averse when the previous outcome had been more favourable and also when the rewarded stimulus was the 8 kHz tone as opposed to the 2 kHz tone.

**Table 2:** $\Delta$iBIC scores for computational models of judgement bias decision data.

Model Parameters	$\Delta \hbox{iBIC}$
$C_{p/r}$, $\lambda$, $\sigma$, $\omega$, $\beta$	0
$\lambda$, $\sigma$, $\omega$, $\beta$	10.604
$\lambda$, $\omega$, $\beta$	41.397
$C_{p/r}$, $\lambda$, $\omega$, $\beta$	100.980
$\delta$, $C_{p/r}$, $\lambda$, $\sigma$, $\omega$, $\beta$	106.018
$\sigma$, $\omega$, $\beta$	147.211
$\delta$, $C_{p/r}$, $\lambda$, $\omega$, $\beta$	148.596
$\delta$ , $\lambda$, $\sigma$, $\omega$, $\beta$	164.567
$\delta$, $\sigma$, $\omega$, $\beta$	176.784
$\delta$, $\lambda$, $\omega$, $\beta$	185.755
$C_{p/r}$, $\sigma$, $\omega$, $\beta$	270.717
$\delta$, $C_{p/r}$, $\sigma$ , $\omega$, $\beta$	325.151
$\omega$, $\beta$	554.897
$C_{p/r}$, $\omega$, $\beta$	619.601
$\delta$, $\omega$, $\beta$	656.009
$\delta$, $C_{p/r}$, $\omega$, $\beta$	838.521

**Figure 4:**
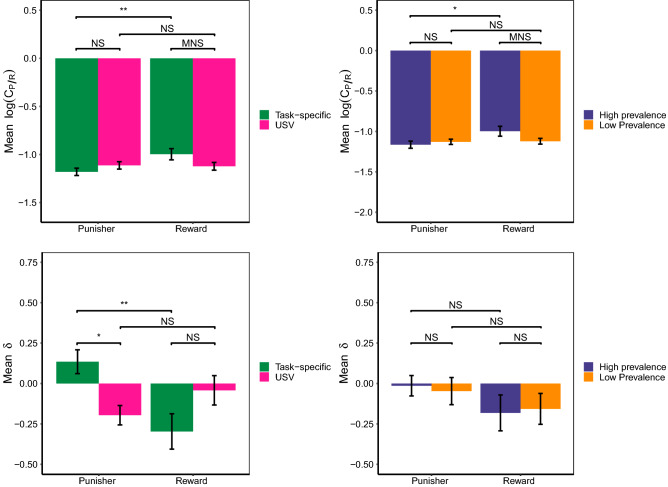
The mean parameter estimates of $C_{p/r}$ (top plots) and $\delta$ (bottom plots) following experience of rewards or punishers that were either task specific or USVs (left-hand plots) and delivered and a high or low prevalence (right-hand plots). Higher values of $C_{p/r}$ reflect a greater weighting of punishers to rewards, and higher values of $\delta$ reflect a greater bias towards the ‘risk-seeking’ response. Error bars represent one standard error.

We subsequently assessed the association between the posterior parameters for $C_{p/r}$ and $\delta$ of the best fitting model and the conditions. The estimates of $C_{p/r}$ for the pre-test high prevalence reward treatment were significantly greater (i.e. greater sensitivity to punishers than rewards) than for the pre-test high prevalence punisher treatment (mean ± SE: $-0.998\pm 0.061$ vs.$-1.164\pm 0.044$, $\hbox{z}=-\,3.005$, $\hbox{p}=0.010$, Fig. 4, Table 3) and tended to be greater for the high prevalence reward treatments than for the low prevalence reward treatment (mean ± SE: $-0.998\pm 0.061$ vs.$-1.122\pm 0.035$, $\hbox{z}=-\,2.238$, $\hbox{p}=0.086$, Fig. 4, Table 3). Additionally, estimates of $C_{p/r}$ for the pre-test sucrose (i.e. task-specific reward) treatment were significantly greater than for the pre-test air-puff (i.e. task-specific punisher) treatment (mean $-0.997\pm 0.058$ SE: ± vs.$-1.180\pm 0.038$, $\hbox{z}=3.334$, $\hbox{p}=0.003$, Fig. 4, Table 3) and the estimates of $C_{p/r}$ tended to be greater for the pre-test sucrose (i.e task specific reward) treatment than for the pre-test 50 kHz (i.e. non-specific reward) treatment (mean ± SE: $-0.997\pm 0.058$ vs.$-1.122\pm 0.040$, $\hbox{z}=-\,2.285$, 0.076, Fig. 4, Table 3). The parameter estimates of $\delta$ also depended on treatment. Specifically, estimates of $\delta$ for the pre-test sucrose (i.e. task-specific reward) treatment were significantly lower (i.e. a more negative bias) than for the pre-test air-puff (i.e. task-specific punisher) treatment (mean ± SE: $-0.297\pm 0.110$ vs.$0.135\pm 0.074$, $\hbox{z}=-\,3.634$, $\hbox{p}=0.001$, Fig. 4, Table 3), and the estimates of $\delta$ were significantly greater for the pre-test air-puff (i.e task specific punisher) treatment than for the pre-test 22 kHz (i.e. non-specific punisher) treatment (mean ± SE: 0.135 ± 0.074 vs.$-0.196\pm 0.060$, $\hbox{z}=2.786$, 0.020, Fig. 4, Table 3). The remaining comparisons tested were not significant (Fig. 4, Table 3). Thus, our results suggest that the greater risk-aversion induced by pre-test reward compared to punisher experience can be attributed to a greater valuations of punishers than rewards and also a bias towards the safe (i.e. ‘leave’) response.

**Table 3:** Results of pair-wise comparisons of the estimates of $C_{p/r}$ and $\delta$ between different pre-test conditions.

Parameter	Contrast	z-value	p-value
$C_{p/r}$	Task-specific vs. non-specific rewards	− 2.285	0.076
	Task-specific vs. non-specific punishers	− 1.219	0.553
	Non-specific rewards vs. punishers	0.17	0.998
	**Task-specific rewards vs. punishers**	**3.334**	**0.003**
	High vs. low prevalence rewards	− 2.238	0.086
	High vs. low prevalence punishers	0.638	0.898
	Low prevalence rewards vs. punishers	0.129	0.999
	**High prevalence rewards vs. punishers**	− **3.005**	**0.010**
$\delta$	Task-specific vs. non-specific rewards	2.142	0.108
	**Task-specific vs. non-specific punishers**	**2.786**	**0.020**
	Non-specific rewards vs. punishers	− 1.295	0.502
	**Task-specific rewards vs. punishers**	− **3.634**	**0.001**
	High vs. low prevalence rewards	0.197	0.996
	High vs. low prevalence punishers	− 0.266	0.991
	Low prevalence rewards vs. punishers	− 0.880	0.773
	High prevalence rewards vs. punishers	1.343	0.471

Bold values highlight statistically significant results.

## Discussion

Affective state is thought to allow adaptive decision-making by reflecting an individual’s previous experience of rewards and punishers. To examine this putative function of affect, we aimed to answer three questions about the relationship between previous experience of rewards and punishers and current decision-making: (1) Are individuals more risk-seeking in the judgement bias task following experience of rewards compared to punishers and is this dependent on reward and punisher prevalence? (2) Does this effect also depend on the extent to which the rewards and punishers are task-specific? (3) Does this risk-seeking or risk-averse behaviour arise from modulation of the likelihood and/or the value of rewards and punishers?

We found evidence that (1) individuals were not more risk-seeking in the judgement bias task following reward compared with punisher experience but instead were more risk-averse; this effect was dependent on reward and punisher prevalence with a greater effect found between rats that experienced the high compared to low prevalence rewards and punishers (an effect that was marginally non-significant in the model-agnostic analysis but significant in the model-dependent analysis); (2) this risk-aversion was greater when the rewards and punishers were task-specific according to both analytical approaches; (3) the observed judgement bias was found to result from a greater weighting of within-test punishers relative to within-test rewards (i.e. the relative valuation of punishers compared to rewards) in rats with pre-test experience of sucrose compared with pre-test experience of air-puffs. Further support for this finding came from using the progressive ratio lever pressing task, in which rats worked harder for sucrose following pre-test experience of punishers compared to rewards. There was also evidence that it arose from a bias towards the risk-averse response.

Thus, although as hypothesised, judgement bias was modulated by the extent to which an individual’s previous experience was rewarding or punishing, our results do not support the hypothesis that reward experience necessarily induces more risk-seeking decision-making relative to heightened punisher experience. Instead, our results may reflect either that rewards can in fact be more valuable in negative affective states (and therefore more likely to be sought via the risky response), or that the contrast from the prior negative affect induction to the test induced a relatively positive affective state, generating the hypothesized association between this state and a more risk-seeking response profile.

Reward and punisher experience may indeed have induced relatively more positive and negative affective states respectively, but these were expressed in a way that we had not originally hypothesised. An individual experiencing a negatively valenced affective state could be more risk-seeking if they valued rewards more highly or punishers less negatively. Indeed, the model-dependent analysis of data from the judgement bias task revealed that rats that had experienced previous air-puffs weighted present within-test punishers less heavily relative to within-test rewards than those that had experienced previous sucrose. This suggests that previous punisher experience increased the subjective value of the sucrose pellet (and/or decreased the subjective value of the air-puff) relative to previous reward experience.

There is some precedent for this finding: although anhedonia (i.e. a reduced reward valuation) is considered to be a cardinal symptom of depression^26^, greater reward valuation has been observed in individuals experiencing more negative affective states, especially at the mild end of the spectrum. For example, depression in humans has been associated with increased valuation of a number of rewards including food^27^, cigarettes^28^, and alcohol^29^. Similarly, animal studies have demonstrated that short-term stress can result in increased reward valuation^30,31^. It has been suggested this might function to regulate mood by encouraging individuals in poorer mood states to seek mood-enhancing rewards^32,33^. Additionally, Trimmer et al. (2017) suggested that food should be valued more highly in environments in which threats are more frequent as maintaining the high energy reserves needed to evade predation becomes more important^14^.

An alternate explanation is that our results reflect a contrast effect. Some other studies have also found an ‘optimistic’ (i.e. risk-seeking) judgement bias in individuals in affective states that were putatively negative (e.g.^22,34,35^). A commonality between our study and these is the release of individuals from an aversive situation immediately prior to testing. In our study the affect manipulation occurred in a different location to the judgement bias task, and thus the context changed between the previous experience of rewards and punishers and the present test. As demonstrated by contextual fear conditioning, the context of an environment can become associated with emotionally-salient experiences^36,37^. Therefore, it is possible that rats experienced a relief-like state following removal from the environment in which they had been exposed to punishers, and a disappointment-like state following removal from the environment in which they had experienced rewards.

Relief and disappointment would be consistent with the successive negative and positive contrast effects observed in rats and other species; disappointment-like or elation-like responses observed when an individual experiences a downshift or upshift in the availability of a food reward^38–40^. Similarly, recent research with human subjects has demonstrated that self-reported affect can be modulated by the difference between actual and expected earnings (reward prediction error) and not the absolute amount earned^41^. A contrast effect would be most congruous with the model-dependent analysis which revealed that pre-test reward and punisher experience altered both reward and punisher valuation and biased subjects towards or away from the ‘stay’ (i.e. risk-seeking) response, the latter being highly associated with affect^8,15,17^. The finding that reward valuation was enhanced following pre-test air-puff compared to pre-test sucrose experience would also be consistent with the contrast induced affective changes proposed here. Numerous studies have identified an association between negative affect and a decreased reward valuation^11–13,42,43^. This explanation would imply that use of environmental priors can be context-dependent. In particular, an individual may recognise that previous experience might not be relevant in new contexts, and instead rely on the comparison of conditions between contexts to inform behaviour.

Reward and punisher experience within the test session also altered behaviour. Rats were more likely to make the risk-averse response when the previous outcome was more favourable and vice versa; this effect was marginally non-significant in the model-agnostic analysis, and significant in the model-dependent analysis. This could indicate that rats are able to recall the most recent tone and compare whether its frequency is higher or lower than the current tone to deduce the likely outcome.

One could argue that pre-test sucrose consumption could lead to satiation and accordingly to a reduction of sucrose valuation, resulting in apparently risk-averse decision-making independent of changes in affect. However, although satiation could provide a partial explanation for our results, rats will consume far more sucrose pellets within a similar time period given free availability, without showing any signs of satiation and reduction in motivation to feed. The maximum number of sucrose pellets a rat could receive in the pre-treatment and test session combined was 36. Previous research has shown that rats will consume 50 sucrose pellets in an average of 5 min^44^. Additionally, during training for the judgement bias task, rats consumed 48.4 ± 3.2 (mean ± SE) sucrose pellets in the 15-min training session on the day prior to discrimination training (i.e. prior to experiencing the air-puff), and 57.4 ± 2.4 sucrose pellets in the 30-min training session on the day prior to their first test session.

Moreover, if satiation did underlie our results it might be expected that rats would be increasingly risk-averse as the session progressed and more sucrose was consumed, but our analysis found no time-dependent changes in judgement bias. Similarly, as the trials are self-initiated, the latency to initiate a trial following sucrose delivery, as a proxy of consumption time, can be examined. If rats that had received sucrose prior to testing were sated, we would expect that the duration of their self-determined inter-trial intervals following sucrose delivery would be increased. However, there were no significant treatment differences in the duration of the self-determined inter-trials interval following sucrose delivery. There were also no treatment differences in duration of the inter-trial intervals following air-puff delivery which would counter the possibility that habituation to the air-puff in rats pre-exposed to the air-puff underlies our results.

Satiety following sucrose consumption and habituation to air-puffs are also inconsistent with the model-dependent analysis which revealed that greater risk-aversion resulting from the pre-test experience arose from both an increased sensitivity to outcome punishment versus reward and a bias towards the safe response. If satiety or habituation did underlie our results, we would only anticipate treatment-dependent variation in the parameter characterising reward and punisher value (i.e. relative sensitivity to rewards and punishers, $C_{p/r}$) which was not the case. Likewise, habituation to air-puff cannot explain the results of the lever-pressing task in which there was only a rewarding outcome.

This study also revealed that decision-making depended on the task-specificity of previous experience. While there was a difference in judgement bias between rats exposed to task-specific rewards and punishers (i.e. ones that were the same as those used in the judgement bias task; pellets and air-puffs), there was no difference between rats exposed to the rewarding and punishing ultrasonic vocalisations (USVs). This is contrary to the findings of Saito et al. (2016)^45^, in which a difference in judgement bias was observed between rats that were presented with 22 kHz and 50 kHz calls. The simplest explanation for this result is that the USVs were not as rewarding or punishing as the sucrose and air-puff, and consequently exerted a much weaker effect on judgement bias. Additionally, and unlike the study by Saito et al. (2016), the rats in this study were not housed in social isolation, which may have also reduced the salience of the USVs (as a result of increased exposure to USVs and social interaction in the home cage). However, this interpretation is not congruent with the findings from the progressive ratio lever pressing task (PRLP task) in which the effect of manipulation valence was not found to depend on manipulation task-specificity. A potential explanation for these findings could be that, unlike responses in the PRLP task which appeared to be domain-general in its influences (i.e. influenced similarly by both punishers and both reward types) prediction of outcomes in the judgement bias task was influenced by specific previous experience only. The possibility that previous reward or punisher experience may have both general and specific effects on different kinds of decision-making is supported by human neural imaging studies which have demonstrated that there is only partial overlap of prediction error signals during learning with different reward and punisher modalities^46,47^. The observed interaction between manipulation valence and task-specificity thus raises the question of whether judgement bias is sensitive to the task-specificity of the affect manipulation. Although numerous studies have found that affect manipulations unrelated to the task influence judgement bias, further investigation is needed to determine to what extent the congruity of previous experience to within-task decision-making modulates judgement bias.

It is notable that variation in judgement bias could be attributed to both a ‘bias’ towards or away from the ‘risk-averse’ response, and also differences in the relative weighting of punishers and rewards. The study thus implicates an important role of reward and punisher valuation in judgement bias. Accordingly, reward and punisher valuation should be considered during experimental design and the interpretation of results of judgement bias studies. The modelling approach used here allowed more effective analysis of data by combining together information across trials to concentrate the strength of the results; and so results which were marginally non-significant in the model-agnostic analysis (i.e. the effect of previous outcome; and the pairwise comparison between high prevalence rewards and punishers) were then significant in the model-dependent analysis. Consequently, this study highlights the strengths of a computational approach; both in allowing dissection of the processes underlying judgement bias and increasing power to detect treatment effects.

Both the model-dependent (in which the parameter characterising the effect was only fitted at the subject-level—i.e. was the same across treatments for the same subject) and model-agnostic analyses revealed a strong effect of which of the tones were rewarded and punished on judgement bias; rats were more risk-seeking when the 2 kHz compared the 8 kHz tone was rewarded. This result was also observed by Jones et al. (2018) and might reflect that the probe tones are perceptually closer to the 2 kHz than 8 kHz tone^25^. This should be investigated in future studies.

In summary, although reward and punisher experience altered decision-making (via the relative weighting of rewards and punishers and a risk-averse or risk-seeking bias), the affective states that the manipulations induced is unclear. The rats in the sucrose condition could have experienced a relatively positive affective state compared with the rats in the air-puff condition, with the observed shift in judgement bias reflecting an uncommon but nonetheless observed affect-related change in the subjective valuation of rewards or punishers. However, it is also possible that the contrast between the manipulation and test environment induced a relatively negative affective state in the rats that had experienced pre-test sucrose compared to pre-test air-puffs. This would suggest that affect not only depends on previous experience but also the context of such experience.

This study therefore highlights the general conceptual problem in this field of identifying what affective state an animal is actually in when investigating and validating measures of animal affect and welfare. Even when using an operational definition that links presentation of rewards and punishers to positive and negative affective state, differences between environments in which the rewards and punishers are experienced and the test situation itself may generate contrast effects that influence affect during tests. Utilisation of affect manipulations that are active during the tests themselves (e.g. alterations to the test environment such as bright light^48^; drugs) and home-cage testing^16^ offer ways around this problem. Nevertheless, a recent meta-analysis which demonstrated a link between pharmacologically-induced negative affect and pessimistic decision-making also identified considerable heterogeneity amongst study findings^17^ indicating that links between presumed within-test affective states and decision-making are not always straightforward and in line with the general hypothesis that positive and negative affective states generate ‘optimistic’/risk-seeking and ‘pessimistic’/risk-averse biases respectively. One possible explanation suggested by this study is that affective influences on reward valuation and predicted decision outcomes can be disentangled and may sometimes be in opposition, hence generating unexpected results. In future work, computational methods will allow a better understanding of the way in which these factors influence decision-making processes and how they relate to affect and welfare.

## Methods

### Subjects

Subjects were 16 male Lister Hooded rats (Charles River, Margate, UK). Two rats did not complete judgement bias (JB) testing due to poor performance in training; they did not reach the progression criterion at the discrimination stage of training (see: Jones et al. (2018)^25^). The rats were housed in stable pairs in cages measuring 560x340x190mm and kept under a 12-h reverse light cycle, with lights off at 7am and on at 7pm. Two cardboard tubes and an aspen block were provided as enrichment and subjects had ad libitum access to food (LabDiet) and water. This research received ethical approval from the Animal Welfare and Ethical Review Body at the University of Bristol (UIN: UB/16/004) and adhered to ASAB/ABS guidelines for the use of animals in research. Subjects were rehomed as pet rats on completion of the experiment.

### Procedure

Subjects first completed training and testing on the progressive ratio lever pressing (PRLP) task, before being trained and tested on the JB task. Prior to each test session, rats received rewards and punishers at a high or low prevalence. Once rats had met the criterion to progress to testing for each task, test sessions occurred twice per week, on a Tuesday and Thursday, with additional training sessions on Monday, Wednesday, and Friday. Rats were not food or water restricted prior to training or testing.

### Apparatus

Four identical operant boxes measuring $508\times 254\times 305\hbox{mm}$ placed in acoustic isolation chambers were used for both the JB task and PRLP task. A wall divided each box into two sections measuring $254\times 254\times 305\hbox{mm}$. Training and testing of each task occurred in only one of these sections. During the PRLP task, a retractable lever was attached to the centre of the end wall of each operant box, and a plastic pot was attached to a pellet feeder was placed adjacent to this lever. For the JB task, the retractable lever was replaced with a pellet/air-puff delivery trough, the pot was removed, and a speaker was placed on top of each operant box (facing down). The pre-test treatment took place in one of two identical operant boxes, both measuring $305\times 178\times 355\hbox{mm}$ in separate isolation chambers in separate rooms adjacent to the testing room. A plastic pot was attached to the end wall in both boxes. This pot was attached to a pellet feeder in one box, and an ultrasonic speaker was placed in the chamber containing the other box. The operant equipment was manufactured by Coulbourn Instruments (Allentown, PA, USA), and operated by Graphic State (v4) software. The ultrasonic vocalisations (USVs) were broadcast using Avisoft software and ultrasound gate hardware (Avisoft Bioacoustics, Berlin, Germany). The sucrose pellets used throughout the experiment were Bioserv (Frenchtown, NJ, USA) Dustless Precision Pellets (45 mg).

### Judgement bias task

The JB task followed the methodology of Jones et al. (2018)^25^. JB task trials were self-initiated by the rat inserting their snout into the food delivery trough which led to a tone being played. Rats were trained to keep their snout in the trough for 2 s when the positive tone played to receive a sucrose pellet, and remove it when the negative tone played to avoid an air-puff. Removal of the snout before 2 s had elapsed resulted in no sucrose or air-puff being delivered regardless of the tone presented. The reference tones had a frequency of 2 kHz or 8 kHz, and the positive tone was 2 kHz for half of the rats and 8 kHz for the remaining half. In test sessions, three probe tones (2.8 kHz, 4 kHz, 5.6 kHz) were presented on six trials each in addition to the reference tones on 21 trials each. As tonal frequency adheres to Weber’s law and is perceived on an approximately logarithmic scale^49^, these tones were selected as they are equidistant on a log-scale hence allowing the perceived differences between the tonal intervals to remain approximately constant. The order of tone presentation was randomised and the ambiguous tones were non-reinforced.

### Progressive ratio lever pressing task

To train rats to press a lever to obtain sucrose, each lever-press resulted in the lever retracting for 1 s and a sucrose pellet being delivered into the pot. An autoshaping procedure ran simultaneously, such that every 60 s the lever would retract and a sucrose pellet would be delivered, even if the lever had not been pressed, after which the lever became re-available^50,51^. Initially, the duration of the training sessions was 15 min. Once subjects had completed at least three 15-min training sessions and had made at least 15 lever presses in a session, the duration of training sessions was increased to 30 min. Rats progressed to testing following completion of two 30-min training sessions in which at least 30 lever presses were made. During testing, the number of presses required before the sucrose pellet was delivered increased in increments of five lever presses, starting at one lever press. The test session ended after 30 min had elapsed.

### Affect manipulation

Reward or punisher experience was manipulated in the 15 min prior to each JB task and PRLP task test session. We adopt Rolls’ (2013) definition of rewards as anything that an animal will work for, and a punisher as anything that an animal will attempt to escape from or avoid. The rewards delivered were one sucrose pellet or playback of 50 kHz USVs lasting 8 s, and the punishers delivered were an air-puff directed towards the rat that was stopped as soon as the rat moved away or playback of 22 kHz USVs of 8 s duration. Rats will work to hear playback of 50 kHz USVs, which are emitted by rats in situations considered to be affectively positive, and will work to avoid playback of 22 kHz USVs which are emitted by rats during unpleasant experiences^24^. Similarly, rats will work to obtain sucrose pellets, and will actively escape air-puffs^52–54^. Each reward or punisher was delivered at either a high or low prevalence. The reward or punisher was delivered at 15 randomly selected times in the high prevalence condition (i.e. an average frequency of once per minute), and once at a randomly selected point during the low prevalence condition (i.e. an average frequency of once per 15 min). The experimental design was within-subject and therefore each rat experienced eight manipulations of reward or punisher experience per task over four weeks. Rats experienced the same reward or punisher at different prevalences within the same week, the order of rewards or punishers experienced was unique for each rat on each task, and this order was assigned randomly. Each rat experienced the high prevalence condition on the first test day of the week for half the test weeks (selected randomly for each rat). Except for the air-puff, which was administered by the experimenter using an air-cannister (Fellowes, Doncaster, UK: Air Duster 300ml), delivery of the rewards and punishers was automated. The 50 kHz USV playback and 22 kHz playback were recorded from rats unknown to the experimental subjects. The 50 kHz vocalisations were induced by placing the rats back into their home cage following a brief period in a neutral holding cage. The 22 kHz USVs were induced by placing the rats in an open arena.

### Data analysis

Data analysis comprised two approaches: a model-agnostic (statistical) and model-dependent approach. Decision data from the JB task were analysed using both approaches which assessed whether there were treatment differences in JB (model-agnostic analysis) and explored the potential cognitive processes underlying these treatment differences (model-dependent analysis). Data from the PRLP task were only analysed using a model-agnostic approach to assess whether there were treatment differences in the number of lever presses made in a session. These data were unsuitable for analysis using a model-dependent approach as each test session provided only one data point (i.e. number of lever presses).

#### Model-agnostic statistical analysis

The response variables analysed were: the total number of lever presses in the PRLP task, and the decision to ‘stay’ (risk-seeking) or ‘go’ (risk-averse) in the JB task. These data were analysed using generalised linear mixed models (GLMM) implemented in the lme4^55^ and nlme^56^ packages in R^57^. Where models assumed a Gaussian error function, the residuals of each model were visually inspected to verify that the model assumptions of normality of error and homogeneity of variance were met. Following log-transformation of the total number of lever presses, these assumptions were not violated in any model. Likelihood-ratio tests (LRT) were used to assess whether the difference in model deviance was significant when a parameter was removed from the model.

These GLMMs included a random effect of individual and session, with session nested within individual. All models included the following fixed effects: manipulation valence (reward or punisher), task-specificity (non-specific or specific), manipulation prevalence (high or low), and interactions between manipulation valence, task-specificity, and prevalence. Additional fixed effect terms included in the model of decision were: a variable which coded whether the 2 kHz or 8 kHz tone was used as the rewarded test stimulus, number of trials completed prior to the decision, the tone presented, the favourability of the previous outcome (treated as a continuous variable; where 1 = sucrose, 0 = nothing, − 1 = air-puff), and the interaction between stimulus and valence. We verified that treating previous outcome as a discrete rather than continuous variable makes no qualitative difference to the results. To investigate significant and marginally non-significant interaction terms in the model, post-hoc comparisons were conducted using a simultaneous pairwise Tukey procedure for general linear hypotheses within the R package multcomp^58^.

In addition to the above GLMMs which were used to examine our core research questions, we fitted GLMMs to the self-determined inter-trial intervals (i.e. the time between exiting and re-entering the trough between trials) following sucrose or air-puff delivery. These GLMMS included a random effect of individual and session, with session nested within individual. The predictor variables were the interaction between manipulation valence, prevalence, and task-specificity, as this would highlight any treatment differences across the eight treatments, and the interactions between manipulation valence and task-specificity and manipulation valence and prevalence, as these were identified as significant (or marginally non-significant) predictors of judgement bias. Both GLMMs included a term to control for the effect of trial, and terms to control for lower order effects of manipulation valence, task-specificity, and manipulation prevalence.

#### Model-dependent analysis: judgement bias

We used a Bayesian-decision theoretic model to characterise decision-making in the JB task^21,59^. On each trial of the task, subjects are presented with a tone (*s*) which we consider to take values between $-2$ and 2, where $-2$ represents the punished reference tone, 2 represents the rewarded reference tone, and 0 is equidistant from the reference tones and is thus entirely ambiguous. The subject’s perception of the tone, *x*, will inform their estimation of the probability that the tone signals the delivery of a punisher, assuming that $s<0$ is associated with a punisher, and $s>0$ with a reward:

$$\begin{aligned} P(\text{pun}|x)&=\int _{-\infty }^0 ds\ P(s|x) \end{aligned}$$

The expected value of making the ‘stay’ response depends on both the probability that the tone presented (*s*) signals a test punisher, given the subject’s perception of the tone (*x*) and the subject’s subjective value of the test sucrose and air-puff, $c^{+}$ and $c^{-}$ respectively:

$$\begin{aligned} E_{p/r}(x)=c^+(1-P(\text{pun}|x))-c^-P(\text{pun}|x) \end{aligned}$$

The expected reward for making the ‘go’ response is 0. Thus, assuming that the posterior probability of *s* given *x* follows a Gaussian distribution with mean *x* and variance $\sigma ^2$, i.e. $P(s|x) \sim{\mathscr{N}}(s: x,\sigma ^2)$, it is optimal for a subject to make the ‘stay’ response under the following condition:

$$\begin{aligned}&c^+(1-P(\text{pun}|x))-c^-P(\text{pun}|x)>0 \\&c^+(1-\phi _{\sigma }(-x))-c^-\phi _{\sigma }(-x)>0 \\&x>-\phi _{\sigma }^{-1}(\alpha ) \end{aligned}$$

where:

$$\begin{aligned} \alpha =\frac{1}{1+C_{p/r}} \end{aligned}$$

and $C_{p/r}=\frac{c^-}{c^+}$ is the differential sensitivity of the air-puff to sucrose.

The probability that a subject opts to ‘stay’ given the true tone is therefore:

$$\begin{aligned} P_{stay}&=P(E_{p/r}(x)>0|s) \\&=\phi _{\sigma }(s+\phi _{\sigma }^{-1}(\alpha )) \end{aligned}$$

We include four additional parameters: a lapse rate $\lambda$ to account for non-perceptual errors, a bias term $\delta$ to allow biases towards or away from ‘stay’ response which thus provides a representation of risk aversion across all trial types, and a parameter which allows for variation in decision-making within a test session according to the outcome of the most recent trial ($R_{t-1}$), $\omega$, and a parameter which allows for between-subject variation in decision-making according to whether the 2 kHz ($R_s=1$) or 8 kHz ($R_s=-1$) tone was rewarded, $\beta$. The probability of a subject making the ‘stay’ response on any given trial is given as:

$$\begin{aligned} P_{stay}=(1-\lambda )\phi _{\sigma }(s+\phi _{\sigma }^{-1}(\alpha )+\delta +\omega R_{t-1}+\beta R_s)+\frac{\lambda }{2} \end{aligned}$$

A relatively ‘optimistic’ (i.e. risk-seeking) judgement bias can therefore arise from either values of $C_{p/r}$ that are less than 1 or values of $\delta$ greater than 0 . For the purpose of model fitting in which a Gaussian distribution is assumed, the values of $\sigma$ and $C_{p/r}$, which were constrained to positive values only, were log transformed, and the values of $\lambda$, which were constrained between zero and one, were logit transformed.

#### Model fitting

Model fitting was conducted using a four-level hierarchical Bayesian random effects analysis. We assume that the parameters ${\mathbf{h}}^i_j$ for each subject $i \in \{1,\ldots ,M\}$ across test sessions $j \in \{1,\ldots ,N^i\}$ are a random sample from a Gaussian distribution ${\mathbf{h}}^i_j \sim{\mathscr{N}}(\pmb{\mu }^i,\pmb{\Sigma })$ parameterized by $\pmb{\mu }^i$, which is itself a random sample from a Gaussian distribution $\pmb{\mu }^i \sim{\mathscr{N}}({\mathbf{m}},\pmb{\nu })$ parameterized by ${\mathbf{m}}$ and $\pmb{\nu }$ (with diagonal $\pmb{\nu }$). Here, $\pmb{\Sigma }$ is also diagonal, and is another parameter. The parameter $\beta$, which allows decision-making to depend on which of the stimuli is rewarded and punished, is considered to operate solely at the subject level (i.e. fixed across sessions) and so a three-level structure was used when fitting this parameter (i.e. top-level, subject-level, and choice data).

The aim of model-fitting is to identify the parameters which maximize the likelihood of the data, $D=\{D^i_j\}$ for session *j* of rat *i*:

$$\begin{aligned} \pmb{\Sigma }^{ML},{\mathbf{m}}^{ML},\pmb{\nu }^{ML}&\approx \text{argmax}_{\pmb{\Sigma },{\mathbf{m}},\pmb{\nu }}\{P(D|\pmb{\Sigma },{\mathbf{m}},\pmb{\nu })\}\\&=\text{argmax}_{\pmb{\Sigma },{\mathbf{m}},\pmb{\nu }}\left\{ \prod _{i=1}^M \prod _{j=1}^{N^i}\int _{\pmb{\mu }^i}\int _{h_j^i} P(\pmb{\mu }^i|{\mathbf{m}},\pmb{\nu })P({\mathbf{h}}_j^i|\pmb{\mu }^i,\pmb{\Sigma })P(D^i_j|{\mathbf{h}}_j^i)d\pmb{\mu }^id{\mathbf{h}}_j^i\right\} \end{aligned}$$

We estimated these parameters using an expectation-maximisation procedure, which involved iterating between the E and M steps described below until convergence^60^. For the *k*th iteration of the E-step, we use a Laplacian approximation based on the model parameters that maximized the likelihood of the data given the prior distributions (for convenience, not integrating out $\pmb{\mu }^i$, as would be possible under the current circumstances):

$$\begin{aligned}{\bar{\pmb{\mu }}}^i(k), \{{\bar{{\mathbf{h}}}}^i_j(k)\}=\text{argmax}_{\pmb{\mu }^i,\{{\mathbf{h}}^i_j\}}\left\{ \log P(\pmb{\mu }^i|{\mathbf{m}}(k),\pmb{\nu }(k)) + \sum _{j=1}^{N^i}\log P({\mathbf{h}}^i_j|\pmb{\mu }^i;\pmb{\Sigma }(k))P(D^i_j|{\mathbf{h}}^i_j)\right\} \end{aligned}$$

The inverse Hessian of this maximization leads to *M* covariance matrices, the *i*th of which, ${\mathscr{M}}^i(k)$ has dimensions $(1+N^i)h \times (1+N^i)h$ where *h* is the number of parameters (with rows and columns organized as $\pmb{\mu }^i,{\mathbf{h}}^i_1,{\mathbf{h}}^i_2,\ldots ,{\mathbf{h}}^i_{N^i}$). We write ${\mathscr{M}}^i_{xy}(k)$ for the $x,y^{\text{th}},[x,y\in \{0,1,\ldots N^i\}]$ block of this matrix, with ${\mathscr{M}}^i_{00}(k)$ being the covariance of $\pmb{\mu }^i(k)$, ${\mathscr{M}}^i_{jj}(k)$ being the covariance of ${\mathbf{h}}^i_{j}(k),[j\in \{1,\ldots ,N^i\}]$, and ${\mathscr{M}}^i_{0j}(k)$ being the covariance between $\pmb{\mu }^i(k)$ and ${\mathbf{h}}^i_j(k)$, all on iteration *k*.

The parameters $\pmb{\Sigma }(k)$, ${\mathbf{m}}(k)$, and $\pmb{\nu }(k)$ are then updated in the M-step as follows:

$$\begin{aligned}{\mathbf{m}}(k+ 1)&=\frac{1}{M}\sum _{i=1}^{M}{\bar{\pmb{\mu }}}^i(k) \qquad , \qquad{\mathbf{h}}^i(k+ 1) = \frac{1}{N^i}\sum _{j=1}^{N^i}{\bar{{\mathbf{h}}}}_j^i(k)\\ \pmb{\nu }(k+ 1)&=\text{diag}\left\{ \frac{1}{M}\sum _{i=1}^{M}\left({\bar{\pmb{\mu }}}^i(k) [{\bar{\pmb{\mu }}}^i(k)]^T+{\mathscr{M}}^i_{00}(k)\right) -{\mathbf{m}}(k+ 1)[{\mathbf{m}}(k+ 1)]^T\right\} \\ \pmb{\Sigma }(k+ 1)&=\text{diag}\left\{ \frac{1}{\sum _{i=1}^M N^i}\left[ \sum _{i=1}^{M}\left( \sum _{j=1}^{N^i}{\bar{{\mathbf{h}}}}_j^i(k))[{\bar{{\mathbf{h}}}}_j^i(k)]^T - 2{\bar{{\mathbf{h}}}}_j^i(k))[{\bar{\pmb{\mu }}}^i(k)]^T +{\bar{\pmb{\mu }}}^i(k)[{\bar{\pmb{\mu }}}^i(k)]^T +{\mathscr{M}}^i_{jj}(k) - 2{\mathscr{M}}^i_{0j}(k) +{\mathscr{M}}^i_{00}(k) \right) -N^i ({\mathbf{h}}^i(k+ 1) -{\bar{\pmb{\mu }}}^i(k))[({\mathbf{h}}^i(k+ 1) -{\bar{\pmb{\mu }}}^i(k))]^T\right] \right\} \end{aligned}$$

A generative test, in which set parameter values were used to generate data which was then fitted the model, was conducted to ensure to verify that the model-fitting procedure recovered the parameters appropriately.

#### Model comparison

We compared models according to their integrated Bayes Information Criterion (iBIC) scores:

$$\begin{aligned} \text{iBIC}=N_p\,log\,|D|-2\,log\,P\,(D|\pmb{\Sigma }^{ML},{\mathbf{m}}^{ML},\pmb{\nu }^{ML}) \end{aligned}$$

where $N_p$ is the number of fitted parameters and |*D*| is the number of data points, and $$\begin{aligned}log\,P\,(D|\pmb{\Sigma }^{ML},{\mathbf{m}}^{ML},\pmb{\nu }^{ML}) \end{aligned}$$ is approximated by sampling ${\mathbf{h}}^i_j$ from the empirical prior distribution, calculating $$\begin{aligned}P(D^i_j|{\mathbf{h}}_j^i) \end{aligned}$$ for each sample, averaging these values to approximate $$\begin{aligned}\,P\,(D^i_j|\pmb{\Sigma }^{ML},{\mathbf{m}}^{ML},\pmb{\nu }^{ML}) \end{aligned}$$ and then taking the sum of the logarithms of these estimated probabilities across sessions and individuals.

#### Permutation test and condition comparisons

We used a permutation test to assess whether parameter estimates differed from zero, where this was meaningful. This involved calculating the mean parameter estimate and multiplying randomly selected parameter estimates by minus one and recalculating the mean parameter estimate 10,000 times. A p-value was obtained by calculating the proportion of resampled means with an absolute difference greater than or equal to the observed mean. Comparisons between treatment groups, which were based on the results of the model-agnostic analysis, were conducted using a simultaneous pairwise Tukey procedure for general linear hypotheses within the R package multcomp.
