# Author Correction: Reward and punisher experience alter rodent decision-making in a judgement bias task

**DOI:** 10.1038/s41598-021-00035-w

**Published:** 2021-10-26

**Authors:** Vikki Neville, Jessica King, Iain D. Gilchrist, Peter Dayan, Elizabeth S. Paul, Michael Mendl

**Affiliations:** 1 Bristol Veterinary School, University of Bristol, Bristol, BS40 5DU UK; 2 Animal Sciences Department, University of Florida, Florida, FL 32608 USA; 3 School of Psychological Science, University of Bristol, Bristol, BS8 1TU UK; 4 Max Planck Institute for Biological Cybernetics, Max Plank-Ring 8, 72076 Tübingen, Germany

Correction to: *Scientific Reports* 10.1038/s41598-020-68737-1, published online 16 July 2020

The original version of this Article contained several errors as a result of an incorrect method of model comparison.

In the Methods, Data analysis section, subheading ‘Model comparison’,

"We compared models according to their Bayes Information Criterion (BIC) scores:

$${\text{BIC}} = N_{p} \log |D| - \left( {2\sum\limits_{i = 1}^{M} {\sum\limits_{j = 1}^{{N^{i} }} l } ogP(D|{\mathbf{h}}_{j}^{i} )} \right).$$

where $N_{p}$ is the number of fitted parameters and $| D |$ is the number of data points."

now reads:

"We compared models according to their integrated Bayes Information Criterion (iBIC) scores:

$${\text{iBIC}} = N_{p} \log |D| - 2\log P(D|{{\varvec{\Sigma}}}^{ML} ,{\mathbf{m}}^{ML} , \, {{\varvec{\upnu}}}^{ML} )$$

where $N_{p}$ is the number of fitted parameters, $| D |$ is the number of data points, and $\log P(D|\Sigma^{ML} ,{\mathbf{m}}^{ML} ,{{\varvec{\upnu}}}^{ML} )$ is approximated by sampling ${\mathbf{h}}_{j}^{i}$ from the empirical prior distribution, calculating $P(D_{j}^{i} |{\mathbf{h}}_{j}^{i} )$ for each sample, averaging these values to approximate $P(D_{j}^{i} |\Sigma^{ML} ,{\mathbf{m}}^{ML} ,{{\varvec{\upnu}}}^{ML} )$ and then taking the sum of the logarithms of these estimated probabilities across sessions and individuals."

In the Results section, subheading ‘Model-dependent analysis’,

"When penalizing model complexity (using a form of Bayesian information criterion; BIC), the most parsimonious model included all six of these parameters ($\delta$, $C_{p/r}$, $\lambda$, and $\sigma$, $\omega$, and $\beta$; Fig. 1, Table 2). Across all conditions and subjects, the log-transformed parameter estimates of $C_{p/r}$ were significantly less than zero (mean ± SE: − 1.103 ± 0.023, p < 0.001) while the estimates of $\delta$ were also significantly less than zero (mean ± SE: − 0.100 ± 0.045, p = 0.026)"

now reads:

"When penalizing model complexity (using the integrated Bayesian information criterion; iBIC), the most parsimonious model included all parameters except $\delta$ ($C_{p/r}$, $\lambda$, and $\sigma$, $\omega$, and $\beta$; Table 2). Yet, the estimates from the full model revealed that, across all conditions and subjects, both the log-transformed parameter estimates of $C_{p/r}$ (mean ± SE: − 1.103 ± 0.023, p < 0.001) and the estimates of $\delta$ (mean ± SE: − 0.100 ± 0.045, p = 0.026) were significantly less than zero. Hence, we opted to use the full model for further analysis to gain a more complete picture of the potential decision processes contributing to treatment differences in judgement bias."

As a result of the changes, the values listed in Table 2 have now been calculated using the integrated Bayesian information criterion ∆iBIC rather than ∆BIC.

The original Table 2 and accompanying legend appear below.

**Table 2:** $\Delta$BIC scores for computational models of judgement bias decision data.

Model Parameters	$\Delta \hbox{BIC}$
*δ*, *C*_*p*/*r*_, *λ*, *σ*, *ω*, *β*	0.000
*C*_*p*/*r*_, *λ*, *σ*, *ω*, *β*	6.434
*λ*, *σ*, *ω*, *β*	162.770
*C*_*p*/*r*_, *σ* *ω*, *β*	296.562
*δ*, *λ*, *ω*, *β*	304.763
*δ* *C*_*p*/*r*_, *σ*, *ω*, *β*	307.135
*δ*, *σ*, *ω*, *β*	325.092
*δ* *C*_*p*/*r*_, *λ*, *ω*, *β*	327.474
*C*_*p*/*r*_, *λ*, *ω*, *β*	327.854
*σ*, *ω*, *β*	536.776
*λ*, *ω*, *β*	544.498
*δ*, *C*_*p*/*r*_ *ω*, *β*	999.956
*δ*, *ω*, *β*	1,004.187
*C*_*p*/*r*_, *ω*, *β*	1,158.868
*ω*, *β*	1,418.546

Finally, as a result of a coding error, Figure 1 was incorrect. The graphics showing the ‘Generated’ data have now been revised.

The original Figure 1 and accompanying legend appear below.

**Figure 1:**
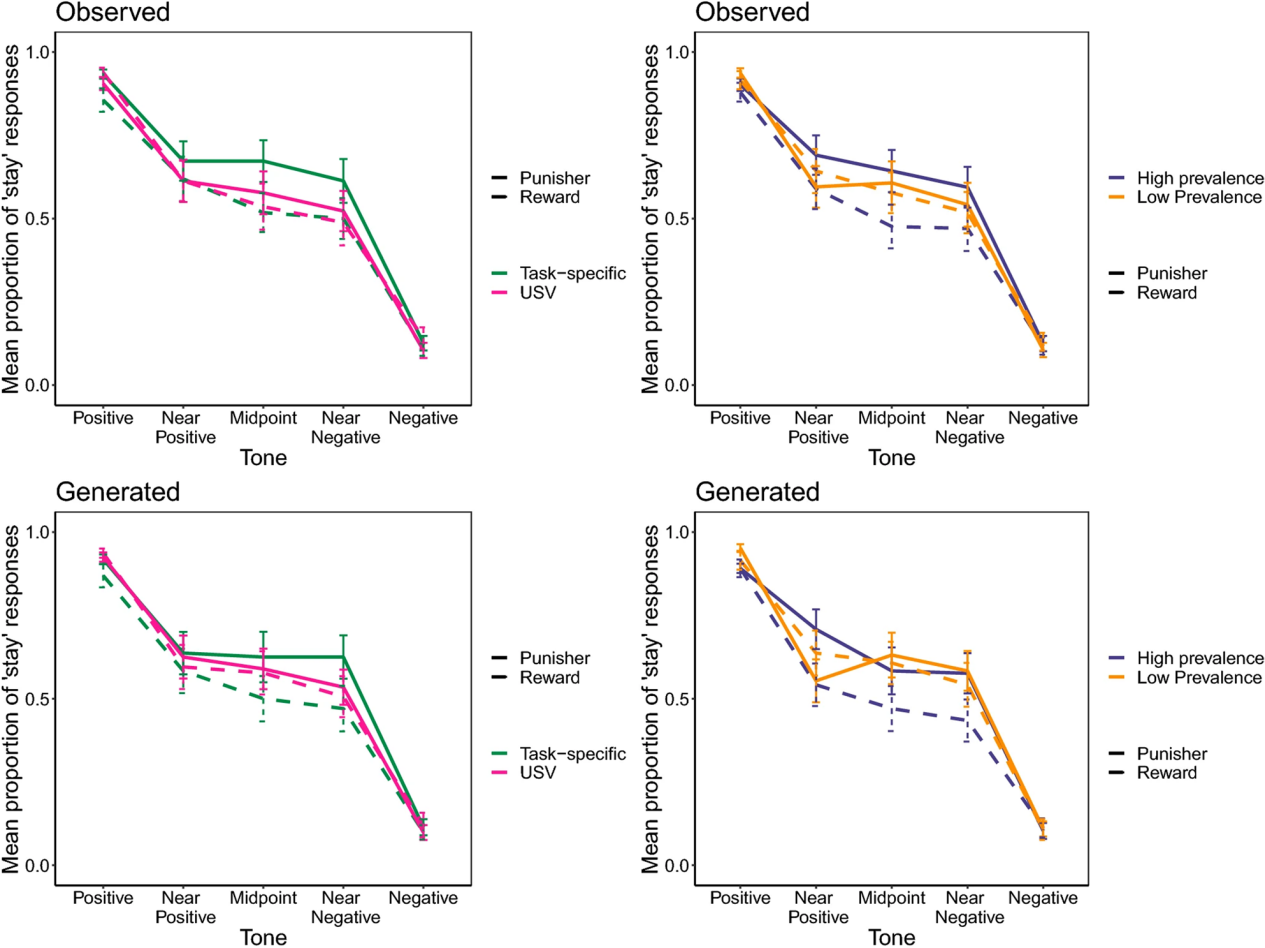
Mean proportion of ‘risk-seeking’ (stay) responses in both the observed and model-generated data split by tone presented following experience of high or low prevalence rewards or punishers and following experience of rewards or punishers that were either task specific or USVs. The top plots show the observed data while the bottom plots show the data generated using the model. The left-hand plots show the data split by task-specificity and manipulation valence (i.e. sucrose—green dashed line; air-puffs—green solid line; 50 kHz USVs—pink dashed line; 22 kHz USVs—pink solid line) and the right-hand plots show the data split by manipulation prevalence and valence (i.e. high prevalence rewards—purple dashed line; high prevalence punishers—purple solid line; low prevalence rewards—orange dashed line; and low prevalence punishers—orange solid line). Error bars represent one standard error.

The original Article has been corrected.

